# Comparative Evaluation of Surface Quality, Tool Wear, and Specific Cutting Energy for Wiper and Conventional Carbide Inserts in Hard Turning of AISI 4340 Alloy Steel

**DOI:** 10.3390/ma13225233

**Published:** 2020-11-19

**Authors:** Adel T. Abbas, Saqib Anwar, Hussien Hegab, Faycal Benyahia, Hazem Ali, Ahmed Elkaseer

**Affiliations:** 1 Department of Mechanical Engineering, College of Engineering, King Saud University, P.O. Box 800, Riyadh 11421, Saudi Arabia; fbenyahia@ksu.edu.sa; 2 Industrial Engineering Department, College of Engineering, King Saud University, P.O. Box 800, Riyadh 11421, Saudi Arabia; sanwar@ksu.edu.sa; 3 Mechanical Design and Production Engineering Department, Cairo University, Giza 12613, Egypt; hussien.hegab@uoit.ca; 4 European X-Ray Free-Electron Laser Facility GmbH, 22869 Schenefeld, Germany; hazem.ali@xfel.eu; 5 Department of Production Engineering and Mechanical Design, Faculty of Engineering, Port Said University, Port Fuad 42526, Egypt; ahmed.elkaseer@kit.edu; 6 Institute for Automation and Applied Informatics, Karlsruhe Institute of Technology, 76344 Karlsruhe, Germany

**Keywords:** AISI 4340 steel alloy, turning operation, cutting parameters, surface roughness, tool wear, specific energy consumption, wiper inserts, conventional round-nose inserts

## Abstract

This paper presents an experimental study into the comparative response of wiper and round-nose conventional carbide inserts coated with TiCN + AL_2_O_3_ + TiN when turning an AISI 4340 steel alloy. The optimal process parameters, as identified by pre-experiments, were used for both types of inserts to determine the machined surface quality, tool wear, and specific cutting energy for different cutting lengths. The wiper inserts provided a substantial improvement in the attainable surface quality compared with the results obtained using conventional inserts under optimal cutting conditions for the entire range of the machined lengths. In addition, the conventional inserts showed a dramatic increase in roughness with an increased length of the cut, while the wiper inserts showed only a minor increase for the same length of cut. A scanning electron microscope was used to examine the wear for both types of inserts. Conventional inserts showed higher trends for both the average and maximum flank wear with cutting length compared to the wiper inserts, except for lengths of 200–400 mm, where conventional inserts showed less average flank wear. A higher accumulation of deposited chips was observed on the flank face of the wiper inserts than the conventional inserts. The experimental results demonstrated that edge chipping was the chief tool wear mechanism on the rake face for both types of insert, with more edge chipping observed in the case of the conventional inserts than the wiper inserts, with negligible evidence of crater wear in either case. The wiper inserts were shown to have a higher specific cutting energy than those detected with conventional inserts. This was attributed to (i) the irregular nose feature of the wiper inserts differing from the simpler round nose geometry of the conventional inserts and (ii) a higher tendency of chip accumulation on the wiper inserts.

## 1. Introduction

Ultra-high-strength steels (HSS), also known as advanced high-strength steels [1], are part of a group of superalloys that are widely used in structural [2,3], military [4], and aerospace industry applications [5], as they show sustainable performance under severe working conditions [6]. In particular, ultra-HSS alloys possess a unique combination of high strength [7], fatigue resistance [8], and ductility [9], which make them prime candidates for applications such as power transmission gears, high-strength bolts, shafts, and airframe parts [10]. The AISI 4340 steel alloy is part of the family of ultra-HSS alloys that are broadly used in military applications [11], which require high-precision machining with tight dimensional accuracy and high surface quality.

However, the relatively poor machinability of ultra-HSS alloys, such as AISI 4340, makes the achievement of high-precision components by conventional machining difficult [12,13]. The superior properties of these materials, such as their high strength, cause rapid tool wear [14] with poor surface quality and inaccurate dimensional tolerances of the machined parts [15,16]. Hence, the machining process becomes costly because it often requires replacing the tool or re-manufacturing or post-processing the workpiece. An alternative is to machine the bulk material in a roughing turning operation, and a subsequent grinding process can be applied to achieve the necessary high precision [17], which increases the cost, wastes time, and reduces productivity [18]. Motivated by the need to find a more efficient process for the machinability of HSS alloys, researchers have investigated precision hard turning [19] with the goal of developing both a tool with the necessary properties and geometries and defining the optimal process parameters required to overcome the high hardness of HSS materials [20].

Previous investigators have examined the machinability of HSS in terms of surface quality [19], cutting force [18], temperature [13], tool wear [16] sustainability aspects [21], and chip control [22], and with different cooling systems, including minimum quantity lubrication (MQL) [23] and cryogenic cooling [24]. Yan et al. [23] studied surface quality and tool wear when machining using MQL. It was revealed that the minimum flank wear and smoothest surface occurred with MQL rather than wet or dry conditions. Li et al. [24] reduced the surface quality and tool wear by applying cryogenic-assisted machining and simultaneously improved the chip-breaking. Shihab et al. [25] showed that PCBN (polycrystalline cubic boron nitride), ceramics, and carbides are suitable tool materials for machining hard-to-cut materials. The use of hybrid machining [26], such as laser-assisted turning [27] and ultrasonic-assisted turning [28], showed excellent performance when machining HSS alloys. García et al. [29] reported that the machinability of S235 carbon steel was improved by 12% with an ultrasonic-vibration-assisted turning technique as a result of the decrease of the specific cutting energy at these high vibration frequencies. Patwari et al. [30] examined the influence of a magnetic field while turning mild steel and found that the surface roughness was improved by 15% compared to conventional turning. To reduce machining costs, wiper inserts are used to improve the surface quality and dimensional accuracy obtained with difficult-to-cut materials [31].

Tool manufacturers have introduced novel wiper inserts, where the nose has a multi-radius geometry to enhance the surface integrity of machined components [32]. From previous studies, the feed rate and nose geometry of the cutting insert were both identified as the dominant factors affecting the final machined surface, with a large radius of the cutting nose reducing the surface roughness [33]. When turning with conventional inserts, low feed rates can produce a better surface quality at the expense of lower metal removal rates and reduced productivity [34]. Wiper inserts were found to be an effective alternative to conventional inserts with large nose radii to improve the surface roughness but with higher feed rates to increase the productivity. Wiper inserts are designed as a series of small radii that combine smoothly to make an effective and efficient nose surface [35]. A number of studies have been conducted on the performance of wiper inserts during hard turning. In particular, for the hard turning of stainless steel 316L [19], oil-hardening non-shrinking steel [36], AISI 4340 steel [37], laser-cladded parts [38], AISI D2 steel [39,40,41], and carbon steel AISI 1045 [42], and in turning 51CrV4 [43].

Researchers have reported that wiper inserts showed an excellent performance in terms of the surface quality at higher rates of feed compared to conventional inserts, though the process parameters [44] and cutting tool geometry [32] did affect the surface quality when using a wiper insert for hard turning. Although using wiper inserts enhanced the surface quality and increased productivity [35], it also showed higher tool rake wear, cutting force, and temperature compared with conventional inserts [17,21]. On the other hand, conventional inserts showed higher flank wear compared with the wiper inserts [13,31]. In addition, a recent work offered an adaptive design model to achieve a balance between the cost, productivity, and quality aspects when using wiper inserts [44].

A number of studies have attempted to address the conflict and produce an optimal trade-off between the obtainable surface quality and tool wear with wiper inserts using optimization techniques [45]. Nevertheless, there has not yet been a detailed comparative evaluation of the multi-responses of wipers and conventional inserts in the hard turning of AISI 4340 steel. In this context, this paper reports an experimental investigation into the relative performance of wiper and conventional round-nose inserts when hard-turning an AISI 4340 steel alloy as a significant input into the discussion on a trade-off between the two inserts. In particular, this study examined the effect of increasing the cutting length on the stability and performance of wiper inserts vs. conventional ones. The quality marks used in this study were surface roughness, tool wear rate, and mechanism and specific cutting energy obtained under the optimal process parameters, as identified by the authors in a previously published paper [11].

This paper is organized as follows. The Material and Methods section is presented first, including the workpiece material and its chemical composition, workpiece shape, experimental setup, insert designations, cutting parameters, and the instrumentation used to characterize the process attributes (surface quality, tool wear, and specific energy). Second, the relative performance of both types of inserts is discussed. Finally, the paper concludes with perceptive insights based on the research findings and proposes recommendations for future work.

## 2. Materials and Methods

The AISI 4340 steel alloy was used in this study. The workpiece material was heat-treated as follows. The material was austenitized at 900 °C for 5 h before it was air-cooled and heated again to 880 °C for 5 h. Next, the alloy was oil quenched, then tempered at 600 °C for 8 h, and finally air-cooled to room temperature. The surface hardness of the workpiece material was assessed and found to be 420 HV. Table 1 lists the chemical composition of the AISI 4340 steel workpiece, which was characterized using a Spectromax metal analyzer by (AMETEX, Boschstr, Germany). A Shimadzu autograph 50 kN servo-electric testing machine (Shimadzu, Tokyo, Japan) was used to conduct the tension tests following the ASTM E8/E8M-16a standard [46] and Table 2 presents its mechanical properties.

**Figure 1:**
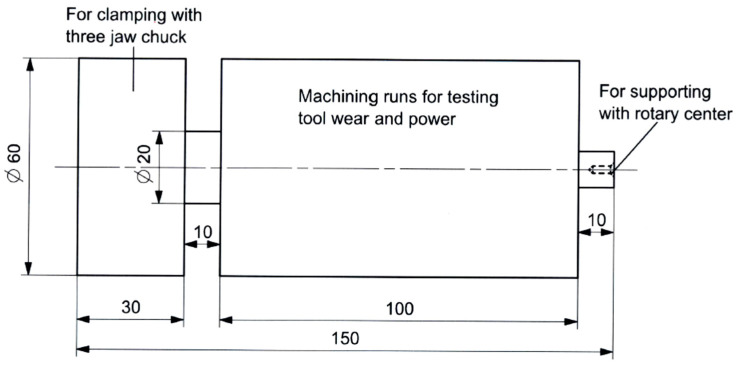
Cylindrical test piece for the turning tests (dimensions are in mm).

The CNC lathe used for the tests was an EMCO Concept Turn 45, equipped with a Siemens Sinumerik 840D (Siemens, Berlin, Germany). The experiments were conducted under flood coolant conditions. For the turning trials, the cylindrical test specimens were 150 mm in length and 60 mm in initial diameter (see Figure 1). These were drilled at one end to produce a standard conical center to support them by the tailstock during the turning tests (see Figure 1).

**Figure 2:**
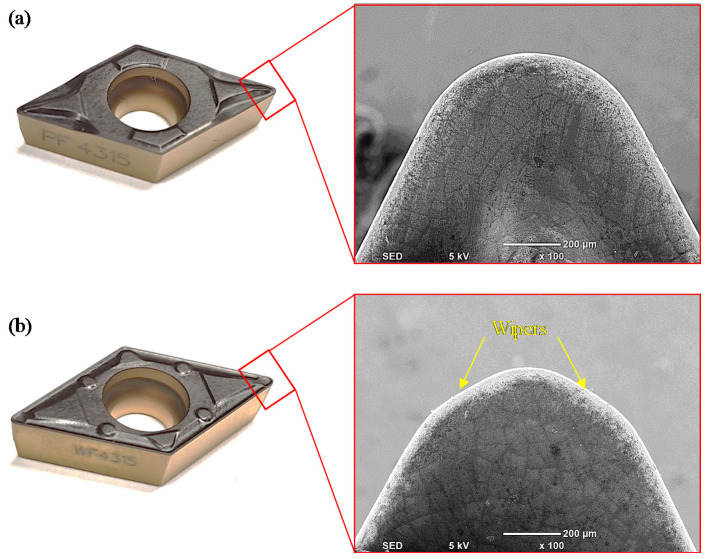
Cutting inserts: (**a**) conventional insert and (**b**) wiper insert.

The machining trials were performed using a Sandvik DCMX11T304-WF Wiper (Sandvik, Stockholm, Sweden) and DCMT11T304-PF carbide inserts (Sandvik, Stockholm, Sweden) (see Figure 2). Both inserts, namely, the wiper and the conventional round nose, were coated using chemical vapor deposition (CVD) on the hard surface with a TiCN + AL_2_O_3_ + TiN coating, and had the same corner radius of 0.4 mm, clearance angle of 7°, and cutting edge angles of 55°, with a rake angle of 6° for the conventional insert and 18° for the wiper insert. Both inserts were fixed into the same tool holder, namely, SDJCL 2020K11 (Sandvik, Stockholm, Sweden), during the turning trials.

The relative performance of both inserts was examined for different cutting lengths: 10, 100, 200, 300, 400, 500, and 700 mm. The investigation was conducted using the ideal cutting parameters for machined surface quality and productivity, as previously identified by the authors [11]. For both the conventional and wiper inserts, the optimal conditions for the lowest surface roughness were as follows: depth of cut (ap) = 0.1 mm and feed rate (f) = 0.05 mm/rev. However, the cutting speeds (Vc) were different: 75 m/min for the conventional insert and 82 m/min for the wiper insert. The first trial was conducted to assess the performance of the two inserts under the almost perfect conditions of new inserts, where this was particularly true for the 10 mm cutting length. The same insert was used for both the 10 mm and 100 mm cutting lengths, whereas a new insert was used for each of the other cutting lengths (a total of six wiper and six conventional inserts). This avoided the uncertainty that could be introduced by taking the insert for the scanning electron microscope (SEM) assessment and re-attaching it. The surface roughness (Ra) was characterized using a Tesa-Rugosurf-90G roughness tester (Tesa, Bugnon, Switzerland). Five measurements were taken for each trial and the average was determined. To measure the power consumed during the cutting trials, two power meters (Tactix 403057, Tactix, Beijing, China) were connected to the power supply of the lathe machine to measure the voltage and the current during the turning of the different samples. Using a balanced three-phase load cutting machine, the power was evaluated by measuring the current (I) on one line, the voltage (V) between two lines and the phase angle (*ϕ*) between the voltage and current. Three readings were recorded during each cutting operation and the total power was calculated using Equation (1):

(1) $$\mathrm{Total}\;\mathrm{power}\;=\;V\times I\times \sqrt{3}\;\cos\;\phi$$

Although the applied cutting conditions for both inserts were quite similar, it was important to normalize the power consumed during the trial to have unbiased comparisons. Thus, after calculating the power consumption, the specific energy was determined by dividing the power by the MRR (material removal rate = Vc × f × ap). A tabletop SEM (JCM 6000Plus, Jeol, Japan) was used to assess the rake and flank faces of the inserts to examine the difference between the wiper and regular round nose inserts. The progressive surface roughness, tool wear, and specific cutting energy for the optimal conditions for the conventional and wiper inserts are listed in Table 3 and Table 4, respectively.

## 3. Results and Discussion

**Figure 3:**
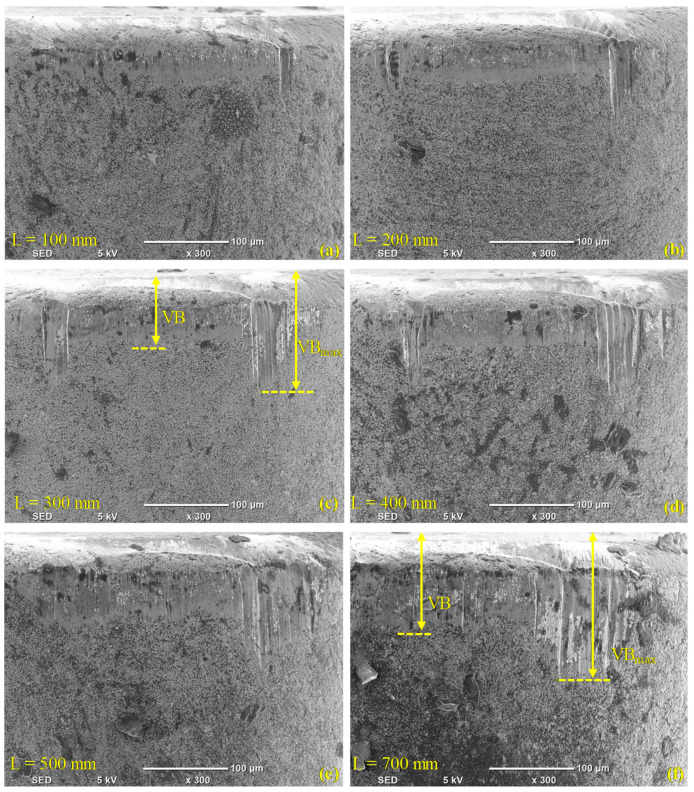
Flank wear in the case of conventional inserts after (**a**) L = 100 mm, (**b**) L = 200 mm, (**c**) L = 300 mm, (**d**) L = 400 mm, (**e**) L = 500 mm, and (**f**) L = 700 mm.

**Figure 4:**
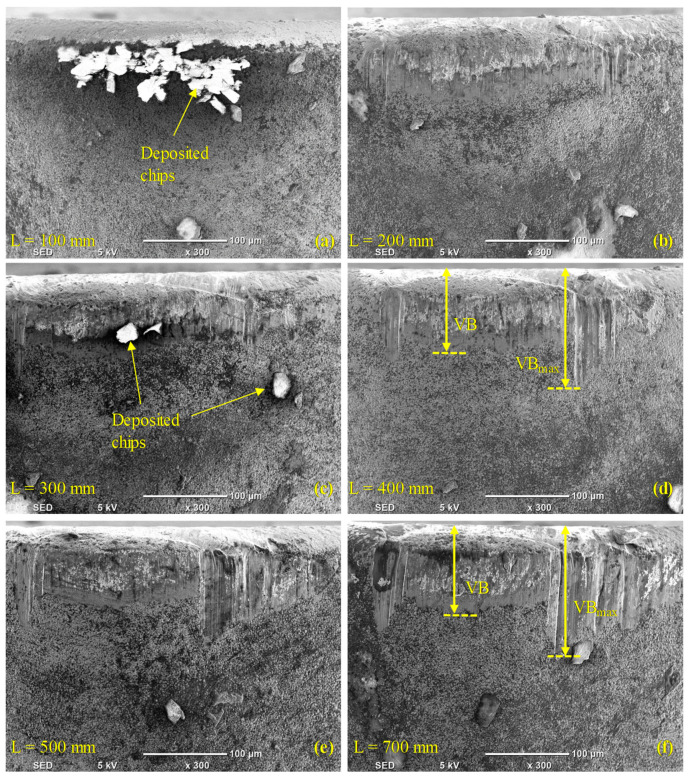
Flank wear in case of wiper inserts after (**a**) L = 100 mm, (**b**) L = 200 mm, (**c**) L = 300 mm, (**d**) L = 400 mm, (**e**) L = 500 mm, and (**f**) L = 700 mm.

Figure 3 and Figure 4 illustrate the morphology (wear) on the faces of the flank of the conventional and wiper inserts, respectively, as assessed via SEM images, and show the progression of the wear for both inserts. In Figure 3a, a VB_max_ of 95.4 µm was detected in the case of the conventional insert after turning a length of 100 mm. In contrast, there was negligible flank wear (36.7 µm) in the case of the wiper insert (see Figure 4a), where only a few chips were deposited near the cutting edge. Overall, a higher accumulation of chips was found on the wiper inserts’ flank face compared to the conventional inserts. Zhang et al. [38] also observed higher accumulation at the built-up edge in the case of the wiper inserts. This was ascribed to the higher cutting forces exerted by the wiper inserts during turning, which can be explained by the thicker chip load due to the irregular geometry of a wiper insert. However, the insert geometry meant that the chips were mostly deposited on the relief flank face and did not make further contact with the workpiece; therefore, they did not adversely affect the workpiece surface finish. The smaller value for VB_max_ produced on the wiper inserts could have also been explained by the shielding effect of the deposited chips. However, the deposited chips near the cutting edge of the wiper insert that formed a temporary built-up edge could lead to an increase in the cutting forces.

**Figure 5:**
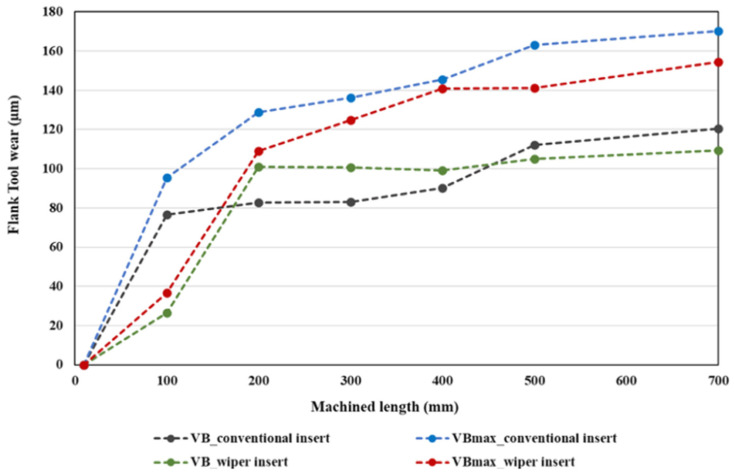
Average (VB) and maximum (VB_max_) flank wear for the conventional and wiper inserts for a range of machined lengths of AISI 4340.

The average flank wear (VB) for the conventional inserts (see Figure 3b) was observed to jump to 76.4 μm in the first 100 mm of machined length, after which, it continuously increased as the machined length increased. For the wiper inserts, the value of VB after machining the first 100 mm was only 26.4 μm, but it rose sharply after machining the next 100 mm to 100.9 μm, after which, it remained stable at around the same value until the machining length was 700 mm, when it increased slightly (see Figure 5). There was a slight reduction in the average flank wear for the wiper inserts at L = 400 mm in Table 4 relative to the cutting length of L = 300 mm. This was because, for each experiment, a different insert was used and some variation can be expected when repeating previous cutting lengths with new inserts. Overall, the conventional inserts showed a higher average and maximum flank wear (see Figure 5). Nevertheless, it is worth emphasizing that although the average wear of the conventional insert was less than of the wiper for the 200 to 400 mm machined lengths (see Figure 5), the total wear over the full 700 mm exhibited by the conventional inserts was larger than those produced by the wiper inserts. Over the studied range of cutting lengths, the conventional inserts showed higher flank wear (VB_max_) for AISI 4340, which is in agreement with previous studies [31,41]. Furthermore, it is worth emphasizing that the wiper inserts operated at a higher cutting speed of 82 m/min compared to 75 m/min for the conventional inserts, and still exhibited lower wear.

**Figure 6:**
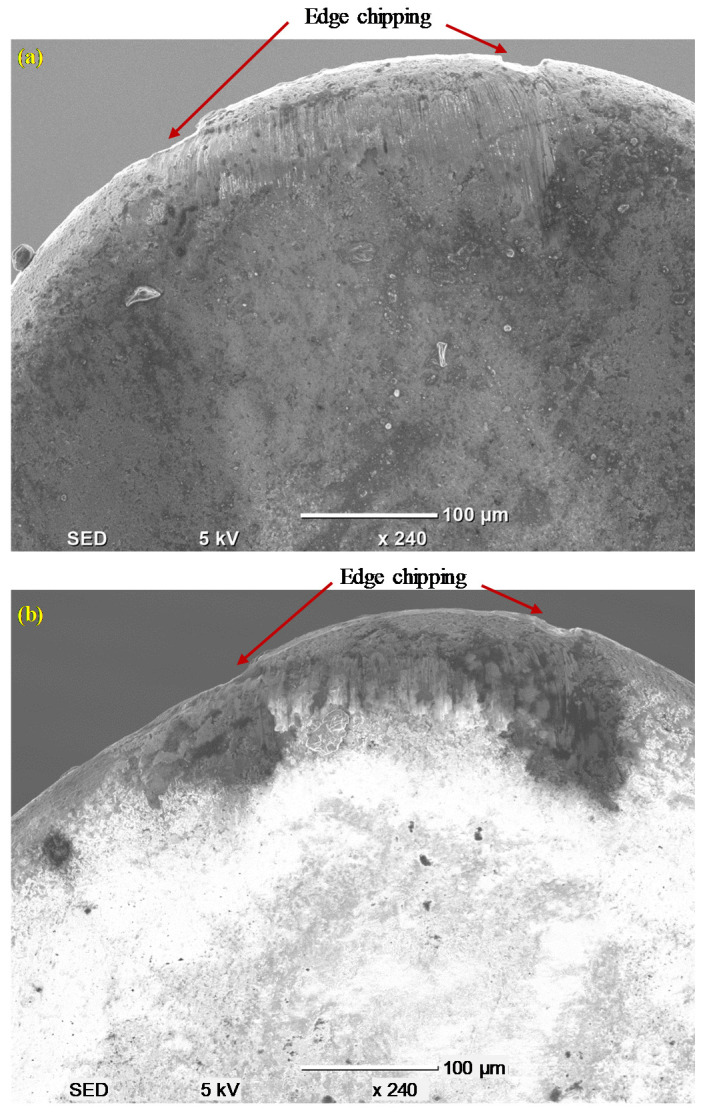
Wear on the rake face of the inserts after L = 400 mm: (**a**) conventional insert and (**b**) wiper insert.

Figure 6 compares the rake wear on the face for the wiper and conventional inserts. Although the inserts had different rake angles (18° and 6°, respectively) and different chip breaker geometries, they exhibited similar wear on the rake face. Edge chipping was the major tool wear mechanism on the rake face for both inserts. Both inserts showed minimal/negligible evidence of crater wear. It seemed that the combination of the lower rake angle (6°) plus high chip breaker geometry in the case of the conventional inserts and the combination of the high rake angle (18°) but lower chip breaker geometry on the wiper inserts resulted in the same wear performance on the rake faces of both the inserts.

**Figure 7:**
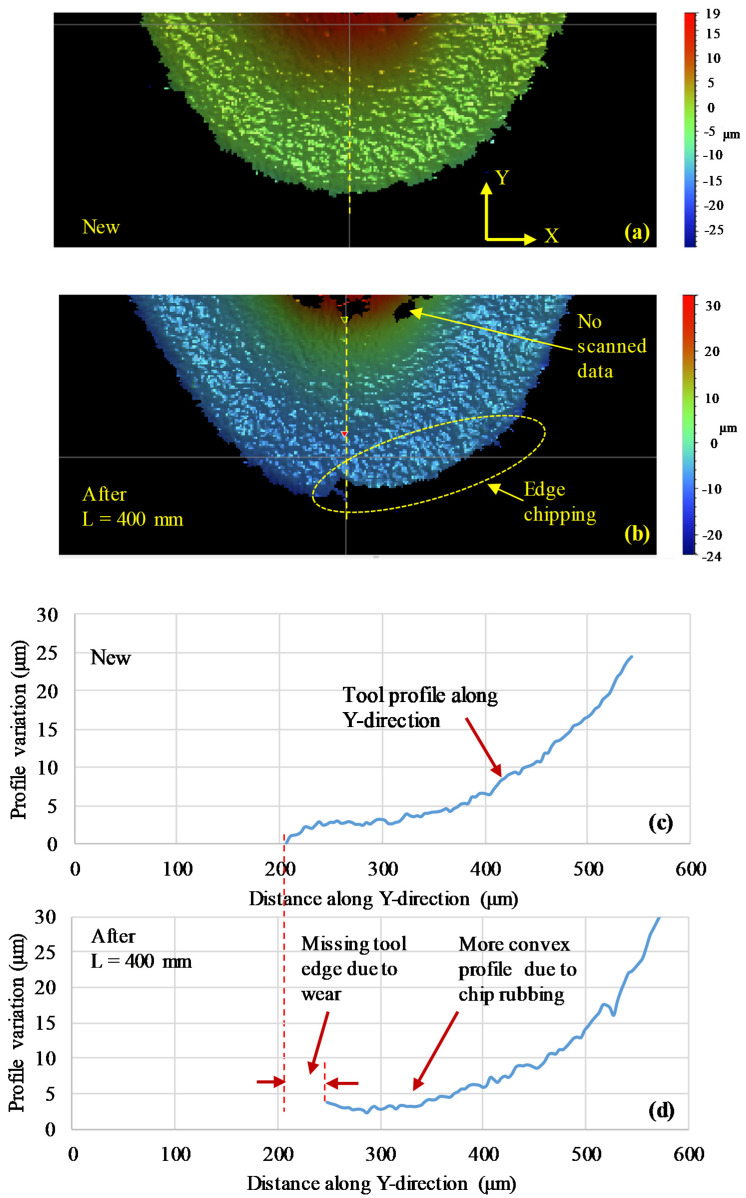
3D scanned surfaces and 2D profiles of the conventional insert before and after use (**a**) 3D surface contour of a new insert; (**b**) 3D surface contour of a used insert after L = 400 mm; (**c**) 2D profile of new insert and (**d**) 2D profile of a used insert after L = 400 mm.

**Figure 8:**
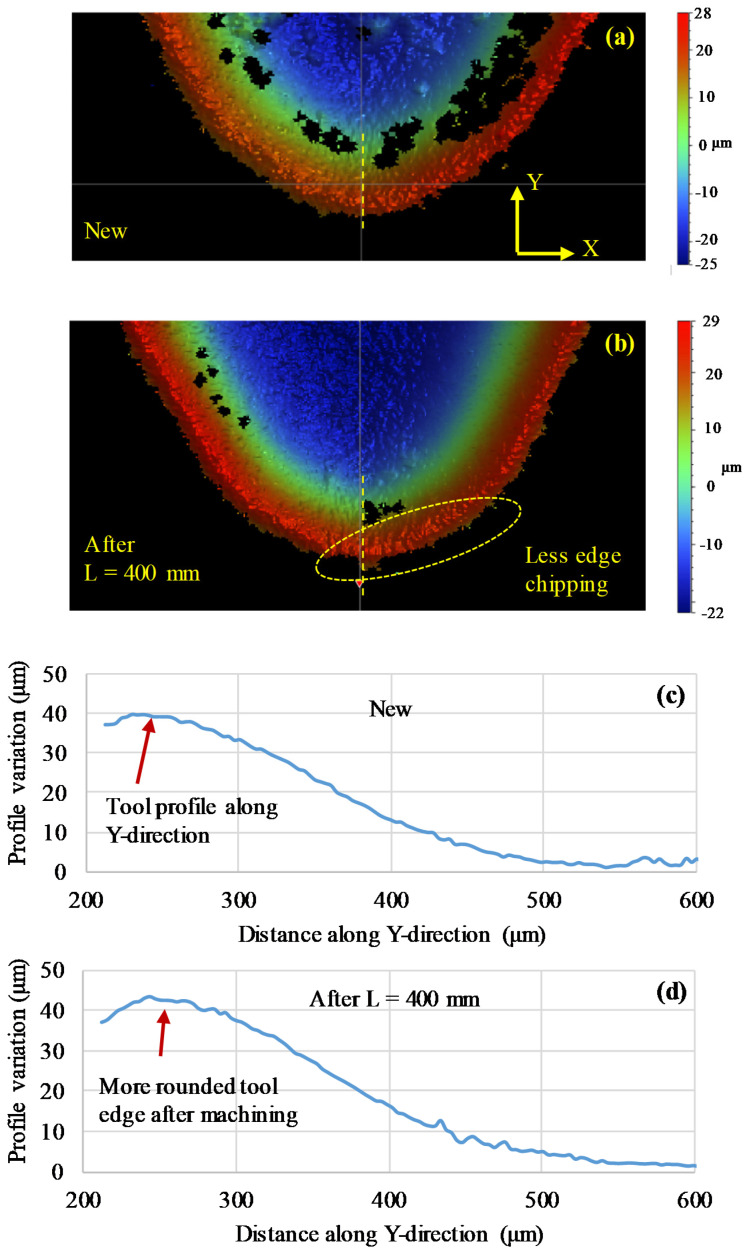
3D scanned surfaces and 2D profiles of the wiper insert before and after use (**a**) 3D surface contour of a new insert; (**b**) 3D surface contour of a used insert after L = 400 mm; (**c**) 2D profile of new insert and (**d**) 2D profile of a used insert after L = 400 mm.

**Figure 9:**
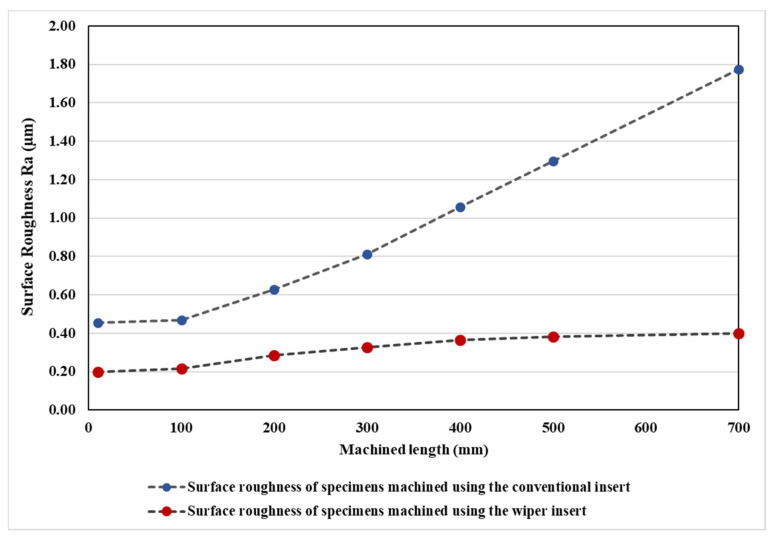
Comparison of the surface roughness for the conventional and wiper inserts at various machined lengths.

To further observe the rake face wear, the geometry of the rake face of the used inserts was scanned using a profilometer (Contour-GTK, Bruker Nano Surfaces Division, Germany) and compared with the geometry of a new insert. Figure 7 presents the profiles of the rake faces of the conventional inserts before and after use. In the 3D scanned contours in Figure 7, some areas on the inserts close to the chip breaker area of the inserts were not scanned due to the limited range of the scanner and the abrupt change of the slope in this region. By comparing the profiles of the new and the used inserts (at L = 400 mm), we see that the profile of the used insert was more convex and steeper and deeper than the new insert, which shows the wear due to the chips rubbing/sliding on the rake face. Furthermore, the tip/edge of the insert was missing due to progressive wear after the machining. Similarly, the profiles of a new and used wiper insert after L = 400 mm are shown in Figure 8. Here, the used wiper insert also showed a more rounded tool edge with a steeper profile compared to the new wiper insert. Moreover, the used wiper insert edge showed a greater height compared to the new insert (see near the arrows in the evaluated profiles in Figure 8), which could be attributed to the built-up edge effect. However, by comparing the 3D images of the scanned tools after L = 400 mm, more edge chipping was observed on the conventional insert, as highlighted in Figure 7. This could be explained by the small rake angle for the conventional insert, which increased the contact area between the insert clearance surface and the workpiece surface associated with higher friction, and accordingly, more chipping. The greater degree of edge chipping of the conventional inserts gave rise to higher surface roughness (Figure 9). However, no noteworthy crater wear could be observed in the scanned profiles for both the inserts.

Figure 9 shows the measured Ra’s for different cutting lengths for both inserts. The tendency for the conventional inserts was to produce a noticeable and increasingly higher level of roughness than the wipers. This is in agreement with the results reported in [11]. The results showed that the surface roughness level produced by the conventional inserts increased by 290% from L = 10 mm to L = 700 mm, while the wiper insert produced a much smaller increase of 103%. The relative improvement of the Ra of the wiper insert increased smoothly from 0.258 μm at L = 10 mm to 1.375 μm at L = 700 mm. This was due to the greater increase in flank wear with an increase in cutting length found in the case of conventional inserts with a detrimental effect on surface roughness, while the wiper inserts showed a relatively lower increase in flank wear (see Figure 5).

**Figure 10:**
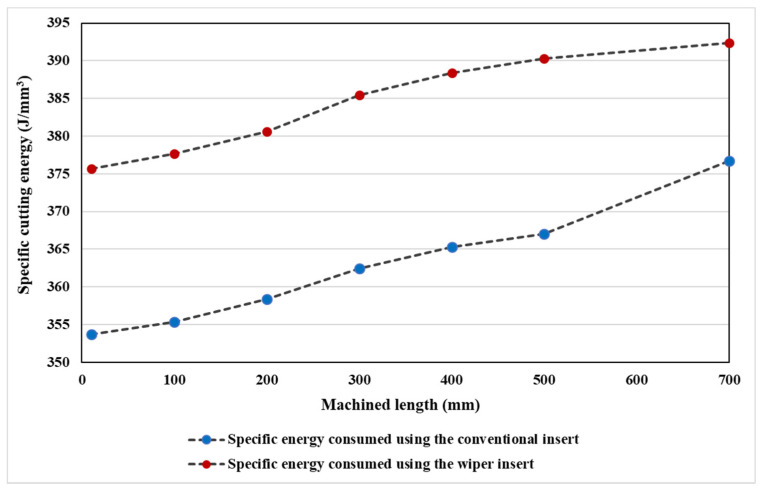
Comparison of the specific energy consumption for the conventional and wiper inserts for various machined lengths.

**Figure 11:**
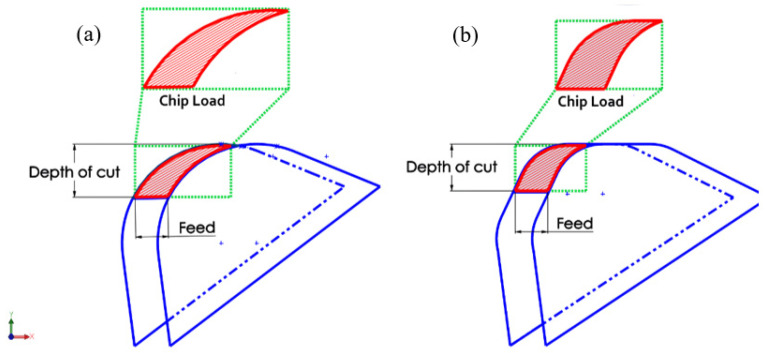
Comparison of the machining action and the geometry of the chip for the (**a**) conventional and (**b**) wiper inserts at the same depth of cut and feed.

A comparison of the specific cutting energy for the conventional and wiper inserts with cutting length is shown in Figure 10. The wiper inserts showed higher specific energy than the conventional inserts due to the thicker chip load (see Figure 11a), while the conventional inserts were associated with a thinner chip load (Figure 11b). Thus, higher cutting forces were developed with the wiper inserts during machining, while conventional inserts exerted less stress, less force, and lower cutting energy [41]. However, it can be noticed in Figure 10 that as the machining length increased from L = 10 mm to L = 700 mm, for the wiper inserts, the specific cutting energy increased by 4.4%, and for the conventional insert, the increase was 6.5%. This means that there was a decrease in the difference between the specific cutting energy between the two inserts from 22.0 J/mm^3^ to 15.6 J/mm^3^, which was a decrease of 29%. This was because the conventional inserts underwent higher progressive flank wear and edge chipping, which led to greater friction between the tool and the workpiece, consequently increasing the power consumption.

## 4. Conclusions and Future Work

The main findings obtained in this work were as follows:

1. With both inserts operating at their optimal cutting parameters, a dramatic reduction in the machined surface roughness of the AISI 4340 steel was obtained when using wiper inserts rather than conventional inserts for the examined range of lengths machined.
2. The longer the length machined, the greater the surface roughness with both inserts, though there was a smaller increase for wiper inserts than for conventional inserts.
3. Conventional inserts always exhibited higher values of maximum flank wear than wiper inserts for all cutting lengths. Regarding the average flank wear, again, the conventional inserts showed higher wear, with an exemption for cutting lengths of L = 200–400 mm, where wiper inserts gave higher values of average flank wear than conventional inserts.
4. It was shown that edge chipping was a major tool wear mechanism on the rake face for both the inserts, with more edge chipping observed with conventional inserts than wiper inserts. However, there was negligible evidence of crater wear for both types of inserts.
5. Higher levels of deposited chip accumulation were observed on the flank face of the wiper inserts compared to the conventional inserts, which could be attributed to the thicker chip load for the wiper inserts.
6. Due to its irregular geometry compared to the conventional round nose, and the higher tendency of chip accumulation, the wiper inserts required a higher specific cutting energy when compared with the conventional inserts.
7. In future work, multi-objective optimization of the turning process of AISI 4340 steel using wiper inserts will be conducted for multi-criterion decision-making. In particular, optimal process conditions for high surface quality, low tool wear, and low specific cutting energy will be identified for a more robust and sustainable turning process.

## Figures and Tables

**Table 1:** Chemical composition of the AISI 4340 workpiece (wt.%).

C	Si	Mn	Ni	Cr	Mo	V	Cu	Fe
0.36	0.12	0.50	2.89	0.96	0.41	0.09	0.08	Balance

**Table 2:** Mechanical properties for AISI 4340.

Properties	Value
Ultimate tensile strength (MPa)	1195
0.2% yield strength (MPa)	1114
Elastic modulus, E (GPa)	206
Reduction in area (%)	59
Elongation (%)	9.3

**Table 3:** Progressive surface roughness, tool flank wear, and specific cutting energy for the optimal conditions for conventional inserts (Vc = 75 m/min, ap = 0.1 mm, f = 0.05 mm/rev).

# Test Run	Edge Type	Cutting Length (mm)	Average Surface Roughness, Ra (μm)	Tool Wear		Specific Cutting Energy (J/mm^3^)
				Average Flank Wear, VB (μm)	Maximum Flank Wear, VB_max_ (μm)	
1	Conventional Inserts	10	0.455	0.000	0.000	353.707
2		100	0.469	76.446	95.455	355.317
3		200	0.628	82.636	128.912	358.390
4		300	0.811	83.049	136.350	362.488
5		400	1.057	90.073	145.440	365.268
6		500	1.295	111.972	163.206	367.024
7		700	1.776	120.588	170.168	376.683

**Table 4:** Progressive surface roughness, tool flank wear, and specific cutting energy for the optimal conditions for wiper inserts (Vc = 82 m/min, ap = 0.1 mm, f = 0.05 mm/rev).

# Test Run	Edge Type	Cutting Length (mm)	Average Surface Roughness, Ra (μm)	Tool Wear		Specific Cutting Energy (J/mm^3^)
				Average Flank Wear, VB (μm)	Maximum Flank Wear, VB_max_ (μm)	
1	Wiper Inserts	10	0.197	0.000	0.000	375.659
2		100	0.215	26.444	36.782	377.707
3		200	0.285	100.919	109.080	380.634
4		300	0.326	100.816	124.780	385.463
5		400	0.365	99.163	140.895	388.390
6		500	0.381	104.948	141.308	390.293
7		700	0.401	109.283	154.430	392.341
